# Silanised Fluoride Hydrotalcites as Functional and Multicomponent Fillers for Dental Composites

**DOI:** 10.3390/biomimetics10060398

**Published:** 2025-06-12

**Authors:** Morena Nocchetti, Michela Piccinini, Antonio Scafuri, Alessandro Di Michele, Valeria Ambrogi

**Affiliations:** 1 Department of Pharmaceutical Sciences, University of Perugia, Via del Liceo, 1, 06123 Perugia, Italy; michela.piccinini@dottorandi.unipg.it (M.P.); antonioscafuri02@gmail.com (A.S.); 2 Department of Physics and Geology, University of Perugia, Via Pascoli, 06123 Perugia, Italy; alessandro.dimichele@unipg.it

**Keywords:** acrylic resins, composites, hydrotalcite, layered double hydroxides, fluoride, silanisation, dental restoration

## Abstract

Acrylic resin composites with a high filler loading, consisting of a fluoride-containing hydrotalcite incorporated into silica nanoparticles, were prepared. The filler was obtained by a multi-step process. First, ZnAl hydrotalcite in fluoride form (HTlc/F) was functionalised with tetraethoxysilane to form Si-O-M bonds (M = Al or Zn) with the brucitic layers. The ethoxysilane groups exposed on the layers were used as nucleation seeds for silica nanoparticles. The composite, named SiO_2_@HTlc/F, was then functionalised with 3-(trimethoxysilyl)-propyl methacrylate groups and used as a filler for acrylic resins. The methacrylate groups on the surface of the inorganic composite participated in the polymerisation process of the resin by minimising the phase separation between inorganic and polymer through the formation of chemical bonds at the polymer–inorganic interface. The filler in the resin increases the degree of polymerisation, bringing it to values very close to 100%. Finally, preliminary studies on the release of fluoride anions showed that they are released slowly over time.

## 1. Introduction

Acrylic resins are materials that have found widespread use in conservative and prosthetic dentistry. Their evolution over time has allowed them to acquire ever-improving performances, making them suitable for multiple applications in all areas of the buccal cavity. Their main positive characteristics are biocompatibility; satisfactory mechanical properties, such as hardness, compressive, and tensile strength, which allow for their use in posterior restorations; and optimal aesthetic properties, which makes them usable also in anterior restorations [1]. However, they suffer from polymerisation shrinkage, and marginal microleakage. To strengthen the polymer and impart additional properties, resin composites have been produced by introducing inorganic fillers. Resin composites are made of two main components. One of these is the resin, which is obtained by photopolymerisation of a mixture of proper monomers constituted by methacrylate derivatives, photoinitiators, accelerators, and photoinhibitors [2]. The other component is an inorganic filler, such as silica, bioactive glass, titanium dioxide, and hydroxyapatite [3,4]. Silica nanoparticles (SiO_2_NPs) have been extensively used as fillers to improve the mechanical properties of acrylic resins. The silica is functionalised with surface modifiers, including 3-(trimethoxysilyl)-propyl methacrylate, to promote interaction between the polymer and filler. The methacrylate groups of the coupling agent participate in polymerisation, reacting with the functional groups of the uncured resin and promoting the formation of chemical bonds between the silica and the polymer [5]. Polymerisation shrinkage of resins has been reduced by filling them with 60 wt% of blends constituted of neat silica and silica nanoparticles silanised with functional silanes of different polarities, as well as neat silica and functionalised silica individually [6]. A reduction in polymerisation shrinkage was found when SiO_2_ functionalised with a quaternary ammonium compound was used as a filler [7].

Functional fillers have been evaluated for their ability to strengthen, inhibit microbial growth, promote self-healing, and remineralise [8,9]. Among remineralising agents, fluoride has been widely studied, as it acts as an anticariogenic agent through different mechanisms, which include the demineralisation prevention and promotion of the remineralisation of dental hard tissues; formation of fluorohydroxyapatite, being less sensitive to an acidic pH; and inhibition of microbe growth and interference with plaque and biofilm growth [10,11]. Therefore, developing a filler capable of providing local and prolonged release of fluoride anions can be a desirable outcome. Hydrotalcite (HTlc) can be designated as a carrier of fluoride anions due to its structural properties. HTlc is a layered material characterised by the general formula [M(II)_1−x_M(III)_x_(OH)_2_](A^−n^)_x/n_.mH_2_O, where M(II) can be Mg, Zn, Cu, etc.; M(III) can be Al, Ga, or Fe; and A^−n^ are the anions, which are located in the interlayer region and balance the positive charges of the layers [12]. Fluoride anions can be easily intercalated into hydrotalcite (HTlc/F) [13,14], and then they can be incorporated in acrylic resins to enable composites to release fluoride anions [15,16,17,18,19,20,21]. The mechanical properties of a commercial light-activated restorative material (Bis-GMA/TEGDM resin containing a glass filler) were improved with the incorporation of 0.7 and 5wt% of HTlc/F. Moreover, the composites exhibited a slow release of fluoride and promoted human dental pulp stem cells’ (hDPSCs) proliferation and differentiation [17]. Indeed, it has been demonstrated that the slow kinetics of fluoride release from dental composites enhances the ability of human dental pulp stem cell subpopulations (STRO-1+ cells) to migrate and differentiate towards an odontoblast-like phenotype [18]. Su et al. demonstrated that commercial composite resins loaded with HTlc/F exhibited the capacity to recharge fluoride [19]. Finally, Bini et al. obtained composites with a load of 10% wt of HTlc/F properly functionalised with an acrylic chain to promote interactions between the resin and inorganic filler at the phase boundary [20]. Composites with multiple biofunctions were prepared by Tammaro et al. by dispersing up to 5% of the HTlc/F-calcium bentonite composite in a commercial restorative resin containing glass filler. The obtained system showed improved mechanical properties, antibiofilm activity, and dental pulp stem cell differentiation [21]. To our knowledge, no papers describe the preparation of silica@fluoride/hydrotalcite hybrids for use as fillers in dental composites.

The aim of this study is to combine functionalised SiO_2_NPs and HTlc/F to obtain a filler (TSM/SiO_2_@HTlc/F, where TSM is 3-(trimethoxysilyl)-propylmethacrylate) for acrylic resins capable of releasing fluoride anions. The first step in this research was the preparation of the filler TSM/SiO_2_@HTlc/F constituted by SiO_2_ nanoparticles grafted onto ZnAl hydrotalcite in fluoride form. Then, this composite was derivatised with methacrylate groups and utilised as a filler at different concentrations for acrylic resins. The obtained composites were characterised by their polymerisation degree and fluoride release.

## 2. Materials and Methods

### 2.1. Materials

Zinc nitrate (Zn(NO_3_)_2_∙6H_2_O), aluminium nitrate (Al(NO_3_)_3_∙9H_2_O), urea ((NH_2_)_2_CO) were obtained from Riedel-de-Haën (Germany); sodium fluoride (NaF) was acquired from Carlo Erba (Milan, Italy). Tetraethoxysilane (TEOS, d = 0.94 g/cm^3^, FW = 208.33 g/mol), anhydrous toluene and concentrated ammonia (28 wt%, d = 0.9 g/cm^3^), 3-(trimethoxysilyl)-propylmethacrylate (TSM, d = 1.045 g/cm^3^, FW = 248.35), bisphenol A glycerolate (1-glycerol/phenol) dimethacrylate (Bis-GMA), bisphenol A ethoxylated dimethacrylate (Bis-EMA), 2-(dimethyl amino)ethyl methacrylate (DMAEMA) were purchased from Sigma-Aldrich Chemical (Milan, Italy). Camphorquinone (CQ) was purchased from Alfa Aesar (Kandel, Germany), and tetraethylene glycol dimethacrylate (TEGDMA) was kindly donated by Esschem Europe (UK). Sodium bicarbonate was purchased from Riedel-de-Haën (Seelze, Germany). The deionised water was obtained by means of a reverse osmosis process using a Milli Q system (Millipore, Rome, Italy). All other reagents and solvents were of the highest degree of purity commercially available.

### 2.2. Synthesis of HTlc in Nitrate Form (HTlc-NO_3_)

HTlc-NO_3_ was prepared according to the urea method [22], using solutions with known concentrations of Zn(NO_3_)_2_∙6H_2_O (0.5 M), Al(NO_3_)_3_∙9H_2_O (0.5 M), and urea (urea/Al molar ratio equal to 4). The volumes of the solutions were chosen so that the molar ratio Al/(Al + Zn) was 0.30. The temperature and reaction time were set at 100 °C and 24 h, respectively. The reaction was conducted under reflux and magnetic stirring. The resulting solid was washed twice with distilled water and dried at 80 °C. The elemental analysis of the sample, performed by Inductively Coupled Plasma-Optical Emission Spectrometry (ICP-OES) and thermogravimetric analysis (TGA), gave the following formula [Zn_0.69_Al_0.31_(OH)_2_](NO_3_)_0.31_ 0.5 H_2_O (ion exchange capacity (IEC) = 2.84 mmol/g).

### 2.3. Preparation of HTlc/F

A weighted amount of HTlc-NO_3_ was equilibrated in 0.19 M aqueous NaF solution (molar ratio F^−^/NO_3_^−^ = 2, mL NaF solution/g HTlc-NO_3_ = 30). The suspension was stirred for 1 day at room temperature [20]. Then, the solid was recovered by centrifugation (4000 rpm for 20 min), washed twice with distilled and decarbonated water, and dried under vacuum over saturated NaCl solution (75% relative humidity). The sample was designated HTlc/F. The fluoride anion content was determined by ion chromatography (IC) and the water content with TGA. The following formula was assigned: [Zn_0.69_Al_0.31_(OH)_2_]F_0.31_·2.5 H_2_O (mmol F/g = 2.22).

### 2.4. Silanisation of the HTlc/F Surface

Before the reaction, all glassware and the HTlc/F are dried overnight in an oven at 100 °C. HTlc/F (472 mg) was dried in a flask under nitrogen flow for 3 h at 70 °C. Then, 15 mL of anhydrous toluene was added, and the mixture was placed under magnetic stirring at 90 °C for 6 h. Then, 120 μL of TEOS (0.54 mmol) was added, and the reaction was maintained under magnetic stirring at 90 °C for 24 h [23]. Finally, the silanisated HTlc/F precipitate was recovered by vacuum filtration, washed three times with anhydrous toluene, and left to dry in an oven at 100 °C for 12 h. The formula of silanisated HTlc/F, determined by ICP-OES, TGA, and IC, was [Zn_0.69_Al_0.31_(OH)_2_]F_0.31_·[Si(OEt)_3_]_0.038_·0.6 H_2_O (mmol F/g = 2.73).

### 2.5. Precipitation of Silica Nanoparticles on Silanisated HTlc/F by Stöeber Method

Silanisated HTlc/F (41.72 mg) and TEOS (0.95 mL; 4.3 mmol) were placed into 22.9 mL of absolute ethanol. The dispersion was stirred until the temperature of 40 °C was reached. Then, 1.6 mL of concentrated ammonia was added, and the reaction was allowed to run for 20 min ([TEOS] = 0.17 M; [NH_3_] = 1 M; [H_2_O] = 2.26 M) [24,25]. The obtained dispersion was centrifugated (15,000 rpm for 10 min) to recover the SiO_2_@HTlc/F. The solid was then dried at 100 °C for 12 h. The elemental analysis of the sample, determined by IC and ICP-OES, gave the following results (expressed as mmol/g): 0.67 Zn; 0.34 Al, 8.84 Si, 0.34 F.

### 2.6. Surface Functionalisation of SiO_2_@HTlc/F with TSM

SiO_2_@HTlc/F (500 mg) and TSM (250 μL, 1.05 mmol) were placed in 50 mL of cyclohexane. The mixture was then sonicated for 1 h. Thereafter, the reaction was stirred under nitrogen flow for 5 h at 70 °C [26]. The solid TSM/SiO_2_@HTlc/F was recovered by filtration, washed three times with anhydrous ethanol and once with deionised water, and dried for 12 h at 80 °C. The content of fluoride in the sample, determined by IC, was 0.14 mmol/g (0.27 wt%).

### 2.7. Synthesis of Acrylic Resin and Composite Resins

The acrylic resin (R) was prepared by adding DMAEMA (1.25 wt% of the total monomer) to a mixture of Bis-GMA (37.5%), Bis-EMA (37.5%), and TEGDMA (25%). The mixture was then ultrasonicated for 30 min; subsequently, CQ, properly grounded in an agate mortar (0.4 wt% of the total monomers), was added [20]. The mixture was then placed into a rectangular mould with dimensions of 0.75 × 1 cm. Then, the mixture was exposed to UV light (type GR.E.400 helios italquartz s.r.l., Milan, Italy) for different times (40, 60, 80, 100, and 150 s). The resulting resins were transferred to Erlenmeyer flasks containing 5 mL of distilled and decarbonated water. The Erlenmeyer flasks, hermetically sealed with screw caps, were placed in a 37 °C water bath for 24 h to facilitate the removal of uncured monomer. Thereafter, the water was eliminated, and the samples were dried at 60 °C for 1 h.

The composites were prepared by incorporating 70, 60, 50, and 40 wt% of TSM/SiO_2_@HTlc/F into the total monomer mixture. The derivatised filler, previously ground in the agate mortar, was added to the polymer mixture, and the obtained mixture was sonicated. Finally, CQ was added, and polymerisation was performed as described above. The composites were labelled as RX (with X = 40, 60, 50, 70, according to the amount of the loaded filler).

### 2.8. In Vitro Release Studies

In vitro fluoride release evaluation from R40 composite was performed on circular samples obtained by placing the mixture (504.37 mg) in a circular mould with a diameter of 2.05 cm. The test was conducted at 37 °C at 100 rpm/min in 50 mL of acceptor fluid composed of a 0.001 M solution of sodium bicarbonate. Withdrawals of 2 mL were collected at predetermined intervals for 14 days, and each withdrawal was replenished with an equal volume of fresh acceptor fluid at 37 °C [27]. Fluoride concentration was determined by IC. The data obtained were expressed as percent release.

### 2.9. Degree of Conversion (DC)

The degree of conversion was determined using the following equation:

$$DC\%=\left(1-\frac{\left(\frac{P_{1}}{P_{2}}\right)\mathrm{polymer}}{\left(\frac{P_{1}}{P_{2}}\right)\mathrm{monomer}}\right)\;\times \;100$$

wherein P_1_ is the peak area of the aliphatic C=C stretching (1635 cm^−1^) and P_2_ is the aromatic C=C stretching peak area (1607 cm^−1^) evaluated in the monomer before polymerisation and in the polymer after polymerisation at different times [28].

### 2.10. Instrumentation

X-ray powder diffraction patterns (XRDs) were taken with a Panalytical X’PERT PRO MPD diffractometer operating at 40 kV and 40 mA, with a 0.017° 2θ step size, and 100 s time per step, using Cu Kα radiation and an X’Celerator detector (PANalytical, Royston, United Kingdom).

TGA and differential thermal analysis (DTA) are obtained by TG-DTA Netzsch STA 490 C thermoanalyser (Netzsch, Selb, Germany) with a heating rate of 10° C/min under an airflow of 30 mL/min.

FT-IR spectra were made in dispersion with KBr using a Jasco model FT-IR-410, Herschl 420 series (Jasco Corporation; Tokyo, Japan) instrument. The spectra were recorded in the range of 400–4000 cm^−1^ with a resolution of 1 cm^−1^ and 100 scans collected. The peak areas were calculated using the spectra in Abs mode. The “2 point base” method was used to draw the baseline. A baseline was drawn between two specified points, and the total area within the baseline was calculated using Jasco Spectra Manager software.

Zinc, aluminium, and silicon content was determined with ICP-OES Varian, Inc. 710-ES series instrument (Santa Clara, CA, USA).

Samples were analysed using Dynamic Light Scattering (DLS) and single particle optical sensing (SPOS) techniques (Zeta potential/particle sizer NICOMP 380 ZLS, Santa Barbara, CA, USA) equipped with a Coherent Innova 70-3 (Laser Innovation, Moorpark, CA, USA) argon ion laser. Samples were properly diluted in ultrapure water before the analysis, measurements were carried out at room temperature, and data were collected for 15 min. The data were expressed as mean diameter using the NICOMP distribution.

The fluoride anions in the samples were determined by IC by a DIONEX DX500 chromatograph (Rome, Italy, correlated with an electrical conductivity detector) using a S4SC column with a NaHCO_3_ solution (3.5 mM) as an eluent and a flow rate of 0.7 mL/s. The fluoride anions in the samples were exchanged for carbonate anions by equilibrating 100 mg of the sample with 10 mL of 0.1 M Na_2_CO_3_ solution for 24 h under magnetic stirring at room temperature. The mother waters containing the exchanged fluorides were submitted to IC analysis.

## 3. Results and Discussion

**Figure 1:**
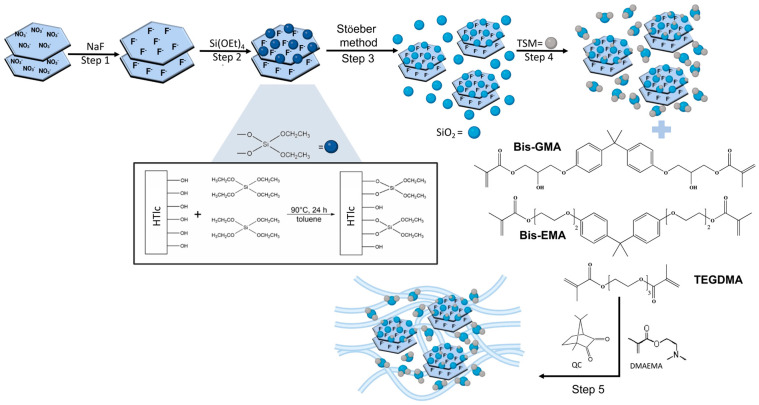
Schematic representation of the preparation of TSM/SiO_2_@HTlc/F and of the corresponding composites.

Figure 1 shows the steps performed for the preparation of the TSM/SiO_2_@HTlc/F, followed by the preparation of the composite resins. The characterisation of the compounds obtained in each step is reported in the following paragraphs.

### 3.1. Silanisated HTlc/F (Steps 1 and 2)

The first two steps of the filler preparation involved the conversion of the as-synthetised HTlc-NO_3_ to the fluoride form and the silanisation of the HTlc/F surface. In detail, HTlc/F was prepared from the corresponding nitrate form by ion exchange, and the surface of the hydrotalcite was functionalised with tetraethylorthosilicate groups. The functionalisation occurs by an intermolecular condensation reaction of at least one of the four ethoxyl groups of TEOS with hydroxyl groups of HTlc, eliminating ethanol and forming M-O-Si bonds (M = Zn, Al) to the HTlc surface [29]. Condensation between HTlc and TEOS has to be carried out in anhydrous toluene and under nitrogen flow to remove humidity, as HTlc is characterised by a high index of hygroscopicity due to its hydrophilic nature. The water molecules, which are bound to the surface, form a “solvation shell” around microcrystals, which can hinder the quantitative progress of the condensation reaction. For this reason, the HTlc was previously dehydrated at 100 °C to remove any surface-bound and co-intercalated water.

**Figure 2:**
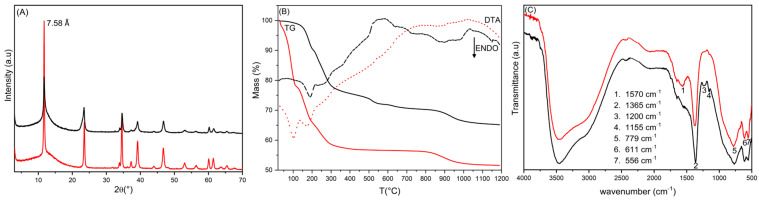
XRD spectra (**A**); TGA (solid lines) and DTA (dashed lines), operating conditions: atmosphere: air, heating rate: 10 °C/min (**B**); FT-IR (**C**) of HTlc/F (red line) and silanisated HTlc/F (black line).

Figure 2A shows the diffraction spectra of HTlc/F before and after the silanisation. The interlayer distance for both materials is 7.58 Å and is consistent with the presence of fluoride anions [13]. For silanisated HTlc/F, no spectral change was observed, indicating that the functionalisation of HTlc occurs only on the surface of the microcrystals. Elemental analysis, performed by ICP (for Zn, Al, Si) and IC (for F), as well as thermogravimetric analysis, confirmed the presence of silicon in the silanisated HTlc/F compound. The data show that the amount of fluoride in HTlc/F is stoichiometric with aluminium, indicating the presence of the sole fluoride phase. During the condensation process, there was no loss of fluoride anions and no variation in the composition of HTlc, as evidenced by the constant Al/F molar ratio and x_Al_.

The TGA and DTA of HTlc/F and the relative silanised product are shown in Figure 2B. The TGA of HTlc/F was characterised by three endothermic weight losses. The initial loss, up to 180 °C (32.5 wt%), is attributable to the elimination of hydration water, corresponding to 2.5 moles of water per mole of hydrotalcite. The subsequent loss, in the temperature range of 180 °C to 700 °C (10.9 wt%), is due to the loss of constitutional water following the condensation of hydroxyl groups; the final loss in the temperature range 750–1200 °C, corresponding to 4.5 wt%, is associated with the loss of the fluoride anion as HF. Elemental and thermogravimetric analyses enabled the determination of the following formula for HTlc/F: [Zn_0.69_Al_0.31_(OH)_2_]F_0.31_·2.5H_2_O. TGA analysis of silanisated HTlc/F shows a lower total weight loss than the original HTlc/F due to the increase in inorganic components and the reduced amount of hydration water. It is noteworthy that, despite the anhydrous conditions under which the hydrotalcite was functionalised, once exposed to air, a certain amount of water was absorbed. Moreover, an additional exothermic weight loss step in the 400–700 °C temperature range, corresponding to 5.1 wt%, is present, and it was attributed to the combustion of the uncondensed ethoxy groups. Coupling the ICP, IC, and TGA data, the following formula for the silanisated HTlc/F sample was obtained: [Zn_0.69_Al_0.31_(OH)_2_]F_0.31_·(Si(OEt)_3_)_0.038_·0.6H_2_O. Moreover, in the aforementioned sample, each TEOS molecule was observed to react with an OH group from the surface.

The formation of siloxane bonds was confirmed by the FT-IR spectrum of silanisated HTlc/F compared with that of HTlc/F (Figure 2C). The spectra share several common features, including a broadened band in the 3000–3700 cm^−1^ region, which is attributed to the stretching of the hydroxyl groups of the M-OH skeleton and the interlayer water involved in forming hydrogen bonds with the hydroxyls of the lamella. Additionally, the bands at 779, 611, and 556 cm^−1^ can be attributed to M-O and M-O-M stretching (with M = Al and Zn); the band at 1365 cm^−1^ can be attributed to traces of carbonate not revealed by other techniques; finally, the band at 1570 cm^−1^ is due to the interlayer water bending mode and it is more intense in the HTlc/F sample due to the higher water content, as observed by TGA. In the FT-IR spectrum of the silanisated HTlc/F, two new bands appear at 1200 and 1155 cm^−1^ and are attributed to the Si-O-Si and Si-O-M stretching modes.

**Figure 3:**
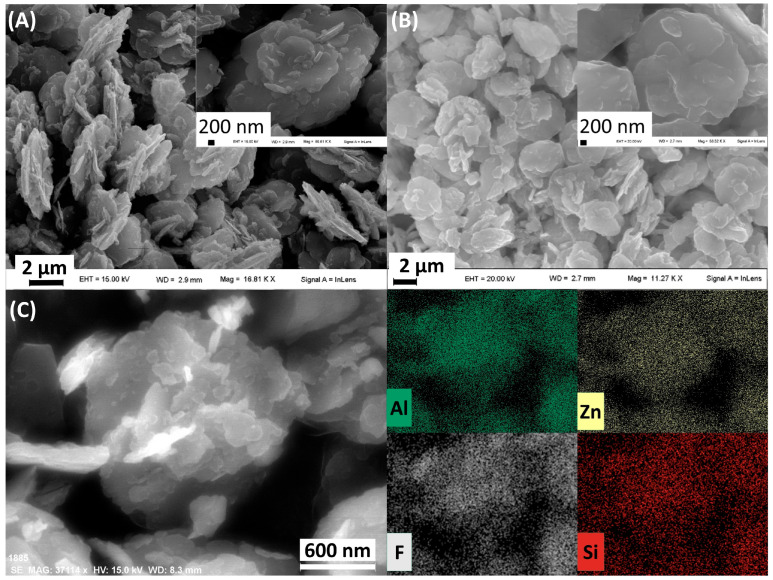
FE-SEM images of HTlc/F (**A**) and silanisated HTlc/F (**B**) at two different magnifications. FE-SEM micrograph of silanisated HTlc/F and EDX images of the metals Al, Zn, F, and Si (**C**).

The morphology of the crystals was investigated by FE-SEM microscopy. Figure 3 compares the microcrystals of HTlc/F before and after functionalisation with TEOS. In HTlc/F, the microcrystals adopt a desert rose structure, with diameters ranging from 1 to 2 μm, formed by aggregates of hexagonal platelet-like particles (Figure 3A). The surface functionalisation process does not induce significant changes in the morphology of the microcrystals, although the surface appears smoother (Figure 3B). The distribution of the elements Zn, Al, Si, and F on the surface of silanisated HTlc/F microcrystals, as obtained by EDX (Figure 3C), provides significant insights. The relative local concentration of each element in the sample is indicated by the brightness and intensity of the colours associated with each element. The distribution of Zn and Al traces the HTlc particles. The distribution of fluoride and silicon follows that of Zn and Al, suggesting a homogeneous silanisation of the HTlc/F surface.

### 3.2. Silica Growth on Silanisated HTlc/F and Functionalisation with TSM (Steps 3 and 4)

In steps 3 and 4, an inorganic composite consisting of HTlc/F and silica (SiO_2_@HTlc/F) was prepared and then functionalised with dangling acrylic groups capable of participating in the polymerisation process. Considering that acrylic resins with very high filler loadings (40–70 wt%) usually exhibit optimal mechanical properties [30] and that content of HTlc/F in the range of 5–10 wt% assures an efficient amount of fluoride [20], the amount of silanisated HTlc/F in SiO_2_@HTlc/F was set at least 14 wt%. Thus, in step 3, silanisated HTlc/F was used as a silica nucleating agent due to the presence of organosilane groups on the HTlc surface. These anchored groups, through M-O-Si bonds to the HTlc surface, are expected to participate in SiO_2_ formation and act as bridges between SiO_2_ and HTlc. Silica precipitation on silanisated HTlc/F was carried out by the Stöeber method, which allows the production of spherical silica nanoparticles with an almost unimodal size distribution and no internal porosity [24]. The Stöeber synthesis involves the hydrolysis of TEOS under basic catalysis conditions. This alkoxide reacts with the water present in the ammonia solution (catalyst) in ethanol (solvent) under constant stirring. Upon contact with water, TEOS undergoes a hydrolysis reaction, resulting in the formation of polymeric silica, according to the following chemical equation: Si(OC_2_H_5_)_4_ + 2 H_2_O → SiO_2_ + 4 C_2_H_5_OH. The basic solution contains a high concentration of hydroxyl ions, which can exchange the fluoride in the HTlc. Therefore, to minimise the fluoride/hydroxyl exchange, silica precipitation was carried out for a short time and with the minimum amount of water corresponding to 2.26 M. According to Bogush, these conditions also make it possible to obtain SiO_2_ nanoparticles around 150 nm in diameter [25].

A stechiometric amount of fluoride with respect to aluminium (molar ratio F/Al = 1) was found in SiO_2_@HTlc/F, and the presence of fluoride was also confirmed by a reflection at 7.58 Å in the XRD pattern of the inorganic composite (Figure S1). The amount of SiO_2_ precipitated was determined considering the mmol/g Si and resulted in 53.8 wt%. The FT-IR spectrum of SiO_2_@HTlc/F compared to that of silanisated HTlc/F is shown in Figure S2, and a detailed description is given in SI. Herein, only the main differences due to the high silica content are reported. In the SiO_2_@HTlc/F spectrum, the band centred at 3250 cm^−1^ appears broader due to the contribution of the silanols present on the surface of amorphous silica. The bands in the 1092–1160 cm^−1^ spectral region, due to the bending mode of the Si-O-Si bond, are increased in intensity, and two new bands at 798 and 946 cm^−1^ appear and are assigned to the antisymmetric stretching mode of the Si-O-Si bond and the stretching of the Si-O bond and the bending mode of Si-O-H, respectively.

**Figure 4:**
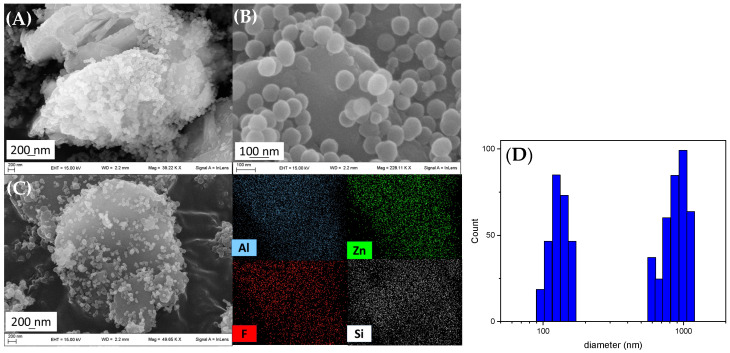
FE-SEM images of SiO_2_@HTlc/F at different magnifications (**A**,**B**) and EDX images of the Al, Zn, F, and Si (**C**). DLS measurement of SiO_2_@HTlc/F (**D**).

The morphology of SiO_2_@HTlc/F was investigated by FE-SEM microscopy. Figure 4A,B shows that a fraction of the SiO_2_NPs with a diameter of about 100 nm are located on the outer surface of the hydrotalcite crystals, confirming the role of silanisated HTlc/F as a nucleating agent for SiO_2_, and the other fraction of SiO_2_ is present as a separate phase. EDX analysis (Figure 4C) shows that the distribution of elements Zn, Al, Si, F on the surface of SiO_2_@HTlc/F is homogeneous. In addition, micrographs show that the SiO_2_NPs have a homogeneous and unimodal particle size distribution, allowing the two inorganic components of the composite to be distinguished by size. To confirm this hypothesis, a DLS (Diffraction Light Scattering) study was performed on the obtained solid (Figure 4D). The size distribution of SiO_2_@HTlc/F shows that the solid is mainly composed of two different size populations: the first, representing 54% of the total particles, has an average diameter of about 1 μm and is represented by silanisated HTlc/F crystals bearing SiO_2_NPs. The second, which represents the remaining 46% of the total particles, is characterised by an average diameter of 129 nm and consists of SiO_2_NPs present as a separate phase. The silica dimensions are consistent with those found by Bogush under the same operating conditions.

The silica present on the SiO_2_@HTlc/F offers free silanols, suitable for the successive functionalisation with acrylate groups. Thus, in the preparation of the inorganic filler, the final step (step 4) was its derivatisation through the formation of covalent bonds between the hydroxyl groups of the silica silanols and the methoxyl groups of 3-(trimethoxysilyl)propyl methacrylate (TSM). This “bonding agent”, alternatively referred to as a “coupling agent”, facilitates chemical interactions with monomers in acrylic resin preparation through the carbonyl-conjugated double bond by participating in the polymerisation reaction of the monomers. The success of functionalisation of SiO_2_@HTlc/F with TSM was confirmed by FT-IR and TGA.

**Figure 5:**
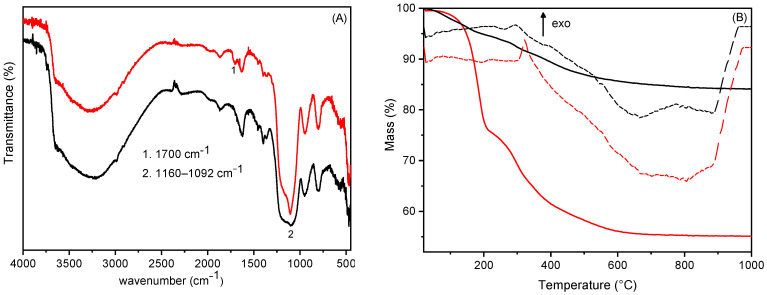
FT-IR spectra (**A**) and TGA (solid lines) and DTA (dashed lines) (operative conditions: atmosphere: air, heating rate: 10 °C/min) (**B**) of SiO_2_@HTlc/F (black line) TSM/SiO_2_@HTlc/F (red line).

Figure 5A displays the FT-IR spectra of SiO_2_@HTlc/F before and after functionalisation with TSM. The spectrum of TSM/SiO_2_@HTlc/F shows a decrease in the band centred at 3250 cm^−1^ due to the reduction of the number of free Si-OH silanols after the reaction with the methacrylic derivative, a narrowing of the band between 1160 and 1092 cm^−1^, attributable to the bending mode of Si-O-Si bonding, and the appearance of a new band at 1700 cm^−1^ due to the methacrylic carbonyl stretching mode.

The weight loss curves obtained from TGA are shown in Figure 5B. The TSM/SiO_2_@HTlc/F showed an additional weight loss of 29% compared to the non-derivatised sample due to TSM decomposition. In addition, the corresponding DTA curve shows the occurrence of an exothermic combustion process in the temperature range 300–500 °C due to the oxidation of the propyl methacrylate moiety. The observed weight loss value of 29% is close to the TSM added in the synthetic procedure (34 wt%); therefore, almost all the moles of 3-(trimethoxy-silyl) propylmethacrylate reacted with the surface silanols.

### 3.3. Preparation of Acrylic Resin Composites Containing TSM/SiO_2_@HTlc/F as a Filler (Step 5)

**Figure 6:**
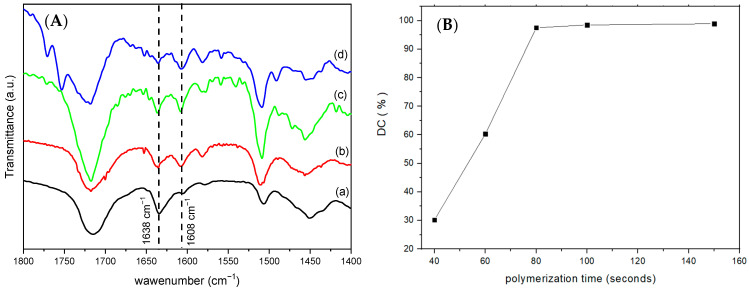
(**A**) FT-IR spectra and (**B**) DC% of R40 at selected curing times: monomer (a), 40 (b), 80 (c), and 150 (d) seconds.

In the preparation of resins for dental use, the evaluation of DC is of fundamental importance. The higher the degree of polymerisation, the lower the amount of free monomer that is toxic at the pulp level [31]. In addition, the DC strongly influences the physical and mechanical properties of the material. The optimum DC will be the one that combines the least amount of free monomer and not excessive stiffness of the material. In a previous work, the DC of monomers (Bis-GMA, Bis-EMA, and TEGDMA) in R was evaluated as a function of time [20]. It was found that a DC of 85.8%, obtained after 70 s of irradiation, made it possible to obtain R with suitable mechanical properties. It was also observed that the presence of a filler strongly affected the DC and then the mechanical properties of the composite resins. Here, to ascertain the maximum inorganic content tolerated by R, a series of composites, R40-R70, containing different weight percentages of inorganics, were prepared. Composites containing inorganic materials in amounts higher than 40% exhibited extreme brittleness. Therefore, the research was focused on R40 and, firstly, on the effect of 40% filler on the monomers DC. R40 composites were prepared by irradiating each side with UV light for increasing times, such as 40, 60, 80, 100, and 150 s. The percentage of double bonds that react and are converted to single bonds during the curing reaction was evaluated by FT-IR (Figure 6). The peak area at 1638 cm^−1^, corresponding to the stretching of the aliphatic C=C double bond, decreases with irradiation time, whereas the peak area at 1608 cm^−1^, corresponding to the stretching of the aromatic C=C double bond, remains unchanged during polymerisation. As illustrated in Figure 6B, there is a direct correlation between the exposure time to ultraviolet light and the DC of R40. The degree of polymerisation increases until a plateau is reached, and the maximum DC value obtained is consistent with the maximum degree of polymerisation observed in commercially available resins (98%) [32]. The R40 obtained with 150 s of irradiation exhibited the optimal degree of conversion. However, this composite was too stiff and brittle, and therefore, the sample obtained at 100 s was selected for further studies due to both its DC, comparable to that of R40 at 150 s and its optimal flexibility degree.

### 3.4. Release Studies

**Figure 7:**
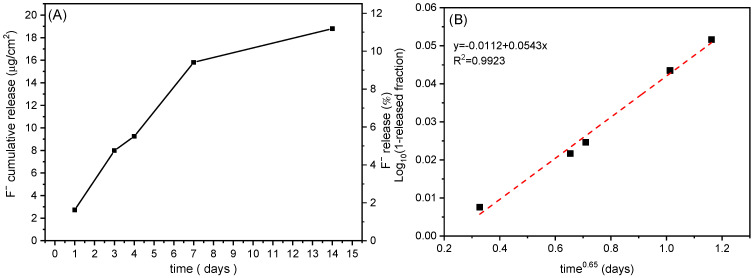
Fluoride released (μg/cm^2^ and %) as a function of time from the R40 cured for 100 s (**A**). Kinetic release curves of R40 cured for 100 s interpolated by Bhaskar model (**B**).

To evaluate the fluoride release profile, an in vitro release study was performed with an R40 cured for 100 s. The release of fluoride ions from the sample was expressed as cumulative release as a function of time (μg cumulative released F^−^/cm^2^ and 100× *g* released F^−^/g total F^−^) (Figure 7).

Figure 7A shows that fluoride ions were released very slowly, and after 14 days, an amount of 18.8 μg/cm^2^ was found, and a plateau was not reached. The slow release can be due to many release steps. First, the ions located on the HTlc surface and at the edges of the lamellae are released. Next, fluoride ions of the interlayer region of HTlc are involved. Since fluoride ions balance the positive charges of the lamellae, their release occurs by ion exchange reaction with anions of the acceptor medium, such as bicarbonate, carbonate, and hydroxides, which diffuse from the outside towards the inside of the composite. Finally, once exchanged, ions diffuse through the composite towards the medium. Xu et al. compared 15 commercial fluoride-releasing restorative materials as glass ionomers, resin-modified glass ionomers, compomers, and composite resins. Glass ionomers and resin-modified glass ionomers showed an initial burst effect of fluoride release (≥40 μg/cm^2^/day), followed by a release decline after the first 3 days. Differently, compomers and composites did not show the burst effect, but the fluoride release was gradual and prolonged for a long time [33]. The same behaviour was shown by R40, and the fluoride release expressed as μg/cm^2^ was comparable with those of most commercial composite resins (6–42 μg/cm^2^ after 14 days of release) described by Xu et al.

The release model which thoroughly describes the release profile of fluoride ions from the composite is that proposed by Boyd et al. [34] for ionic resins, which was also simplified by Bhaskar [35] (Figure 7B). According to this model, when the diffusion through the particle is the rate-limiting step, there is a direct proportionality between log(1-released fraction) and time^0.65^. This method was applied to the obtained data, and from the plot, a good linearity (r = 0.9961) was obtained. This means that diffusion across the HTlc particles is the step limiting the fluoride release [36].

The slow release indicates that the resin is sufficiently water resistant, and this is a desirable characteristic for a material that must be in contact with saliva and remain in place for a long period of time. In addition, the low fluoride concentration prevents a cytotoxic reaction in cell cultures [37], and the prolonged release ensures local remineralisation of teeth over a long period of time. However, despite numerous studies, there is no precise quantitative information on the concentration of fluoride required to prevent caries formation and promote remineralisation [38,39]. On the other hand, if fluoride remains immobilised in the resin [40,41], it may act as an antibiofilm agent. This is an advantage both in restorative dentistry, as it would lead to a reduction in plaque around the restoration, and in removable dentistry, as acrylics are known to promote bacterial and yeast adhesion.

## 4. Conclusions

The aim of this work was to prepare composite dental materials that not only have a restorative or replacement function for compromised tooth structure but also have a preventive role against primary and secondary caries and other oral diseases such as gingivitis and candidiasis. In this work, a method for the functionalisation of hydrotalcites with silane groups was developed. Nanoparticles of silica were precipitated in the presence of HTlc functionalised with silane groups which allowed the formation of Si-O-M bonds (M = Al, Zn of the brucitic layer) between the silica and HTlc. The obtained SiO_2_@HTlc/F joins the typical properties of an anion exchanger and the chemical properties of silica. In fact, the interlayer region of HTlc was used as a medium capable of storing and releasing fluoride anions in a suitable medium. The silica was functionalised with acrylic groups to allow the inorganic component to participate in the polymerisation reaction of the acrylic resin. It is worth noting that the obtained filler had a positive effect on the degree of polymerisation, bringing it to values very close to 100% and allowing it to load the final composite with fluoride anions which were slowly released for an extended period of time.

## Data Availability

The original contributions presented in the study are included in the article; further inquiries can be directed at the corresponding authors.

## Supplementary Materials

The following supporting information can be downloaded at: https://www.mdpi.com/article/10.3390/biomimetics10060398/s1, Figure S1: XRD spectrum of SiO_2_@HTlc/F; Figure S2: FT-IR spectra of silanisated HTlc/F, and SiO_2_@HTlc/F.
