# Supplementary material for: Silanised Fluoride Hydrotalcites as Functional and Multicomponent Fillers for Dental Composites
Source: Biomimetics (Basel). 2025 Jun 12;10(6):398. doi: 10.3390/biomimetics10060398 (PMC12190227; doi:10.3390/biomimetics10060398)
Supplement: Supplementary file 1 [file biomimetics-10-00398-s001.zip › biomimetics-3606971-supplementary.pdf]

SUPPORTING INFORMATION

SILANISED FLUORIDE HYDROTALCITES AS FUNCTIONAL AND MULTICOMPONENT FILLERS FOR DENTAL COMPOSITES

Morena Nocchetti[1]*, Michela Piccinini[1], Antonio Scafuri[1], Alessandro Di Michele[2], Valeria Ambrogi[1]*

[1]Department of Pharmaceutical Sciences, University of Perugia, Via del Liceo, 1, 06123 Perugia, Italy; michela.piccinini@dottorandi.unipg.it (M.P.), antonioscafuri02@gmail.com (A.S.)

[2]Department of Physics and Geology, Via Pascoli, University of Perugia, 006123 Perugia, Italy; alessandro.dimichele@unipg.it (A.D.M.)

*Correspondence: morena.nocchetti@unipg.it (M.N.); valeria.ambrogi@unipg.it (V.A.)

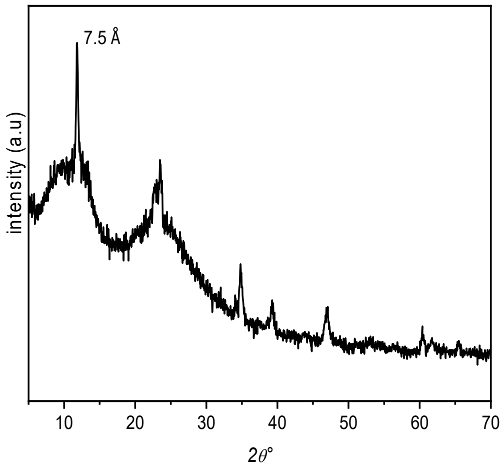

Figure S1. XRD spectrum of SiO_2@HTlc/F dried at 100°C

Figure S2 shows the FT-IR spectra of silanisated HTlc/F and of SiO_2@HTlc/F. In both spectra there is a band between 3000 and 3700 cm^{-1}; which in silanisated HTlc/F is due to the stretching of the M-OH bond, while in SiO_2@HTlc/F it is mainly due to the formation of hydrogen bonds between the hydroxyl groups exposed on the outer surface of the precipitated amorphous silica; it is noted that this band is slightly

broadened after functionalisation with silica, since the contribution of the silanols is greater than that of the M-OH of the HTlc. Another similarity are bands at 1457 and 1365 cm^{-1} corresponding to the stretching of the intercalated CO_3^{-2} ion, whose contribution seems to disappear almost completely in the silanised product due to the dilution of the crystal itself in the precipitated silica. The band at 1630 cm^{-1}, characteristic of the bending mode of H_2O, is present and it is quantitatively more prominent in the silanised product because it is easier to form hydrogen bonds with the free silanols. In the spectral region between 1092 and 1160 cm^{-1}, the bending mode of the Si-O-Si bond is found in spectra of both compounds, but the higher amount of silica in the silanised compound contributes to a significant broadening of this band; also characteristic of the latter are the bands at 798 and 946 cm^{-1}, corresponding to the antisymmetric stretching mode of the Si-O-Si bond and the stretching of the Si-O bond and the bending mode of Si-O-H, respectively. In contrast, the bands at 1200 cm^{-1} are characteristic of silanisated HTlc/F, corresponding to the bending mode of the Si-O-Si bond and 1155 cm^{-1}, related to the stretching of the Si-O-M bond.

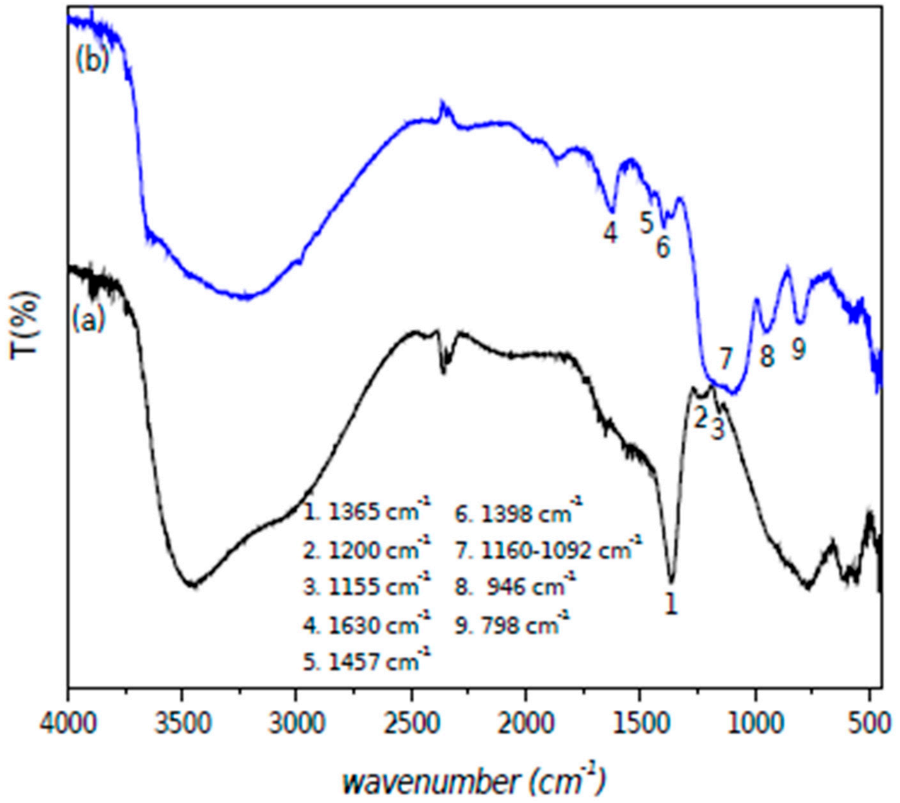

Figure S2. FT-IR spectra of silanisated HTlc/F (a), and SiO_2@HTlc/F (b) dried at 100°C.
